# Darrell R. Abernethy (1949‐2017)

**DOI:** 10.1002/prp2.382

**Published:** 2018-01-09

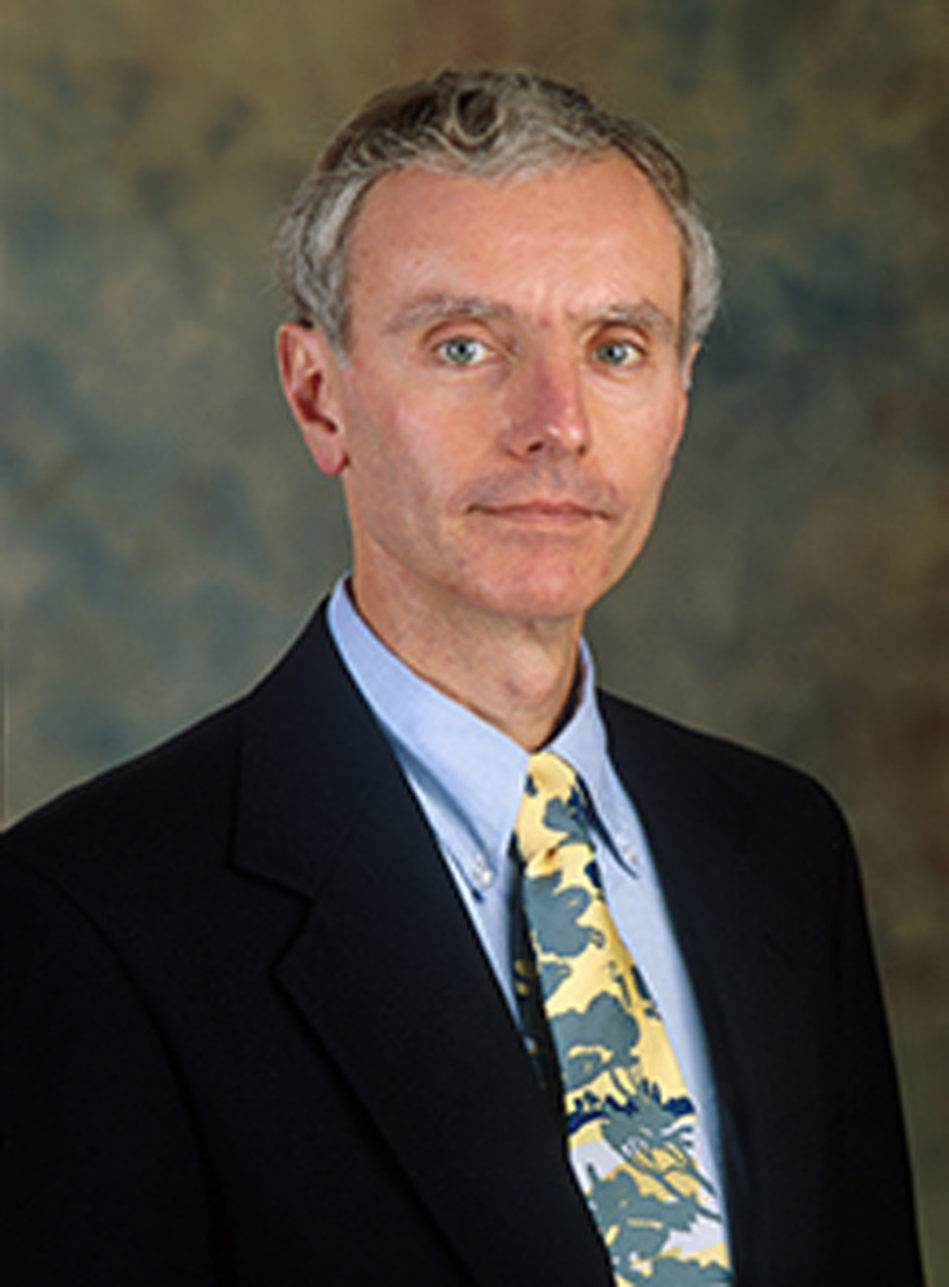

On behalf of everyone at PR&P, I convey the passing of our Editor‐in‐Chief, Dr Darrell Abernethy. Darrell has been part of PR&P since its inception, initially as Deputy EiC and since 2016 as EiC. He was a stalwart of the journal and assisted in the recruitment of a strong editorial board and the communication with editors of its supporter journals. From a professional perspective, Darrell had a distinguished career as a clinical pharmacologist and geriatrician. Darrell completed MD / PhD degrees at the University of Kansas, followed by training in Internal Medicine at the University of Miami (Florida) and in Clinical Pharmacology at Harvard Medical School / Massachusetts General Hospital. He had a number of academic positions and also directed an NIH program regarding the effects of drugs in the elderly. His last appointment was with the FDA where he investigated ways to predict adverse effects of therapeutic drugs. Darrell's excellence was recognized by his peers with numerous awards, including the Nathaniel T. Kwit Memorial Distinguished Service Award from the American College of Clinical Pharmacology and the William B. Abrams Award in Geriatric Clinical Pharmacology and the Rawls‐Palmer Progress in Medicine Award from the American Society for Clinical Pharmacology and Therapeutics.

Darrell contributed back to the discipline at a high level. He was EiC of Pharmacological Reviews and Associate Editor of the Journal of Pharmacology & Experimental Therapeutics as well as Clinical Pharmacology and Therapeutics. This experience was brought to bear during his tenure with PR&P. In addition to his editorial responsibilities and leadership, Darrell was also a leader within the broader pharmacology community being a key contributor to the Clinical Division of the International Union of Basic and Clinical Pharmacology (IUPHAR).

Darrell's major pastimes included sailing and music. He was an affable person and a great encourager of his juniors. He will be sorely missed.

Professor Andrew J Lawrence BSc(Hons), PhD, FBPhS

Current EiC of PR&P

